# Knowledge of Cervical Cancer Screening among Women across Different Socio-Economic Regions of China

**DOI:** 10.1371/journal.pone.0144819

**Published:** 2015-12-14

**Authors:** Jiangli Di, Shannon Rutherford, Jiuling Wu, Bo Song, Lan Ma, Jingyi Chen, Cordia Chu

**Affiliations:** 1 National Centre for Women and Children’s Health, China CDC; Beijing, P.R. China; 2 Griffith University; Brisbane, Queensland, Australia; State University of Maringá/Universidade Estadual de Maringá, BRAZIL

## Abstract

**Background and Objective:**

China has a high burden of cervical cancer (CC) and wide disparities in CC burden exist among different socio-economic regions. In order to reduce these disparities, China’s government launched the National Cervical Cancer Screening Program in Rural Areas (NCCSPRA) in 2009. Understanding the factors associated with underutilization of CC screening among target populations is important to improve the screening participation rate, and a high participation rate is key to achieving the goals of a screening program. However, data on the knowledge of CC among target populations in program areas is lacking in China. This study will investigate the knowledge of CC prevention and control among women in specific project counties to develop a better understanding of factors that might influence CC screening participation in order to improve the implementation of the NCCSPRA.

**Materials and Methods:**

A cross-sectional survey was conducted and face-to-face interview questionnaires were completed by 308 women who received CC screening services in 6 project counties of NCCSPRA across different socio-economic regions of China. ANOVA and Chi-square tests were used to compare the knowledge rates and scores across the different subgroups. Logistic regression was conducted to examine factors associated with knowledge level.

**Results:**

The overall CC knowledge rate of the target population was only 19.5%. Regional socio-economic level, advice from doctors, age, and educational status were strong predictors of knowledge level of CC screening. Significantly lower knowledge rates and scores were identified in older women (55–64 years old), less educated women (with primary school or illiterate), women in less developed regions and women who did not receive any advice about screening results from doctors.

**Conclusion:**

The knowledge of CC screening among women in the project counties of NCCSPRA was found to be very poor. Given the importance of knowledge in encouraging women to participate in screening is key to reducing CC burden in rural women in China, it is urgent that a targeted health promotion intervention is developed and implemented in project counties, especially targeting older women, women with less education and women in less developed regions, and focus on improving their CC knowledge and encouraging them to communicate with health care providers. The health promotion intervention targeting health care providers is also important to improve their knowledge of CC and provide best advice to women.

## Introduction

Cervical cancer caused by sexually-acquired persistent infection with high risk human papillomavirus (HPV), continues to be a global public health problem in the world, and the vast majority of CC deaths occur in women living in developing countries, like China [1]. China’s achievements in reducing incidence and mortality of CC have been significant. The detection rate of CC decreased by 97.8% from the 1950s to the 1990s [2], however, because of its large population, China still contributes about 12% of new global CC cases or deaths [3], and the national incidence and mortality rate have increased by 157.9% and 116.7% from 2003 to 2010 [4–6]. Furthermore, the disease is over-represented in rural areas and less developed regions of China [4–6] owing to inequity in financial resources, infrastructure, health care staff and access to health care [7, 8].

Experience in developed countries has shown that organized high quality screening programs can significantly reduce the number of new cases of CC and the mortality rate associated with them [9, 10]. Beginning in 2009, a free National CC Screening Program in Rural Areas (NCCSPRA), sponsored by government was launched in 221 counties, covering every province and autonomous region [11]. It was the first time that the Chinese government had proposed to widen access in rural areas to CC screening services and it represented a step towards nationwide provision of CC screening [12]. From 2009 to 2011, 11.69 million rural women aged between 35 and 59 years in 221 pilot counties, covering 31 provinces and autonomous regions received this free service [13]. By design, the pilot counties were concentrated in China’s less developed central and western regions. Among the 221 pilot counties, 50% of the counties are in western regions and 35% are in central regions [11]. Beginning in 2012, the screening target age was changed from 35–59 to 35–64 years. The number of women eligible to access the free service increased to 30 million women from rural areas across 1,185 counties in 2012–2014 [14].

The Guideline for Comprehensive CC Prevention and Control [9] mentions that one of the barriers to control of CC is lack of knowledge of CC and its prevention. Many studies from different countries have confirmed that lack of knowledge about CC was a significant barrier to attending CC screening program [15–18], and understanding the factors associated with underutilization of CC screening is important to improve the number of women who attend CC screening [19]. The effectiveness of participation in reducing mortality and incidence has been demonstrated by some research [10, 20]. Hence, the participation rate is one of the most important factors in determining the success of a screening program and women’s knowledge about CC is a very important factor influencing participation in a CC screening program.

A previous study in China showed that the barriers to attending screening included anticipated feeling of anxiety, not knowing the benefits of CC screening, and the possibility of incurable CC [15]. Improving women’s knowledge about CC screening can not only help them to understand and reduce their personal risk of illness, but reduce their anxiety when attending the screening [1]. One study in China suggested that regional socio-economic level was one of the strong factors influencing women’s knowledge towards breast cancer screening in China [21]. We hypothesize that regional socio-economic factors also affect women’s knowledge about CC screening in China. Therefore, our study will explore the knowledge of CC among women in project counties across different socio-economic regions, to identify factors that might influence their knowledge level, in order to improve CC screening attendance.

## Materials and Methods

### Design and sample

The study was a cross-sectional survey of women in Liaoning, Hubei and Shaanxi provinces selected to represent the three regions of China: Eastern, Central and Western China. Eastern China, the most economically advanced of the three regions has an average GDP of US$5464. Central China has an average GDP of US$2630. Western China is the least developed region, with an average GDP of US$2354 [7, 8]. Based on the GDP 2013 figures for Liaoning, Hubei and Shaanxi (US$4410, US$4018 and US$2613, respectively)[22], these three provinces represent the most economically developed, developed, and less developed regions respectively. Within each province, two counties from the NCCSPRA were selected randomly. They are Xunyang and Hanbin in Shaanxi province, Xiangzhou and Zaoyang in Hubei province, Zhuanghe and Wafang Dian in Liaoning province.

Stratified random sampling was used. Towns in each project county were divided into two layers in accordance with the distance away from the county government; a sample town was randomly selected from each layer. A total of 12 sample towns were selected. Women's knowledge rate was selected as the indicator to calculate the sample size required. According to the objective of the NCCSPRA, the expected knowledge rate will be up to 60.0% [14], so the survey sample size formula used is: N = 400 × Q / P = 400 × (1-P) / P ≈ 267. Taking into account other factors, such as invalid questionnaires, the sample size needed to be enlarged by 10%. Therefore, the number of women surveyed was 267 × 1.1 = 294. Since there are 12 surveyed towns, an average 25 women in each town was randomly selected and surveyed.

The data was collected between September and November 2013 using a structured questionnaire. A total of 308 women who received CC screening services of NCCPSA in the six project counties were recruited in the investigation. Among them, 96 were from most developed regions, 106 from developed regions, and 106 from less developed regions. All interviews were conducted face-to-face by trained investigators. The data collection process was frequently checked by a supervisor to ensure the completeness and consistency of the collected information. The purpose of the investigation was explained to the participants. All participants provided their written consent to participate in this study and all were informed about confidentially measures and rights to withdraw. This study was approved by the Ethics Committee of Griffith University, Queensland, Australia (GU Ref No: ENV/33/13/HREC).

### Measures

The questionnaire on knowledge about CC screening was designed through expert consultation and a review of the literature from relevant studies in China and elsewhere [15, 17, 18, 23–28]. It consisted of three sections. The first focused on socio-demographic characteristics, including region, age, nationality, education. The second section assessed the knowledge about CC screening and included 5 subsections: risk factors for CC (7 questions), prevention of CC (7 questions), clinical symptoms of CC (9 questions), benefits of screening (3 questions), and meaning of positive results (9 questions). Most of the questions had only two response options (yes/no or true/false) while some had three response options (yes, no or unclear). This section included 35 questions of equal weight. The section comprised risk factors for CC, CC prevention, clinical symptoms of CC, benefits of screening, and understanding of positive results. Total scores for each section were summed up to determine an overall score (max = 35). The third section included questions about the participants’ results of screening and treatments.

According to the results of the literature review [21], participants with scores of 60% or higher were considered to be knowledgeable. The number of women who achieved this for any one score is defined as the knowledge rate.

### Statistical Analysis

Data were entered into EpiData 3.2 with dual entry verification, consistency, and logic error checking. Statistical analyses were performed by SPSS 22.0. Descriptive analyses were generated for all variables. The ANOVA and *t*-test was used to estimate statistical differences in overall and sectional knowledge scores within the different age groups, educational status and socio-economic regions. The Chi-square test was conducted to compare differences in demographic characteristics between the different regions and in the total and sectional knowledge rates within different age groups, educational status and socio-economic regions.

Logistic regression was performed to examine factors associated with total and sectional knowledge rate, with non- knowledgeable (0) and knowledgeable (1) used as the dependent variable, and the statistical difference factors analyzed by Chi-square test as the independent variables.

## Results

### Demographic Characteristics

A total of 308 (100.0%) valid questionnaires were collected. The average age of the participants was 45.04±8.75 years (range from 23 to 64 years). One hundred and six (34.4%) women were from less developed regions and the same number from developed regions, and 96 (31.2%) from the most developed regions. Most of the participants (57.1%) had junior high school degrees, and 26.6% had only primary school education or were illiterate. Only 5.8% had junior college degrees or higher and 10.4% had graduated from senior/technical school. There was a statistically significant association between the age and educational status (*p*<0.001). All women with junior college or higher degrees were less than 34 years old.

Significant differences were found in the age and the educational levels of the participants among different regions (*p*<0.001); women aged 45–54 years constituted the majority in most developed regions (56.2%) and less developed regions (45.3%). The two most developed regions had the largest representation of women with junior high school degrees (70.8% and 67.9%, respectively) while the less developed regions had the largest representation of illiterate women or those with only primary school education (56.6%). Many of the participants in most developed (39.6%) and developed regions (52.8%) had received advice for screening results from doctors, whereas only 28.3% of the participants in less developed regions got advice from doctors (Table 1).

**Table 1:** Demographic characteristics of women among different regions (n = 308) (From Chi-square test).

Characteristics	Most developed regions (N = 96)	Developed regions (N = 106)	Less developed regions (N = 106)	Total frequency	*χ* ^2^	*p*
Age						
<34	0(0.0)	30(28.3)	2(1.9)	32(10.4)	73.717	<0.001
35–44	28(29.2)	44(41.5)	34(32.1)	106(34.4)		
45–54	54(56.2)	24(22.6)	48(45.3)	126(40.9)		
55–64	14 (14.6)	8(7.5)	22(20.8)	44(14.3)		
Education level						
Junior college or higher	8(8.3)	10(9.4)	0(0.0)	18(5.8)	79.585	<0.001
Senior/Technical school	10(10.4)	12(11.3)	10(9.4)	32(10.4)		
Junior high school	68(70.8)	72(67.9)	36(34.0)	176(57.1)		
Primary school or Illiteracy	10(10.4)	12(11.3)	60(56.6)	82(26.6)		
Received advice from doctors						
Yes	38(39.6)	56(52.8)	30(28.3)	124(40.3)	13.284	0.001
No	58(60.4)	50(47.2)	76(71.7)	184(59.7)		

### Total CC Knowledge Levels

The majority of the participants had heard about CC (288, 93.5%). The average total knowledge score among all participants was 12.8±8.00, out of a possible range of 0 to 35, with 60 (19.5%) women reaching the knowledgeable mark. Analyses by Chi-square test revealed a significant difference in the total knowledge rate among women with different educational levels, in different regions and who had received advice from doctors or not (*p*<0.001) (Tables 2, 3 and 4). The results of ANOVA and *t*-test also showed there were statistically significant differences in the total knowledge scores among women with different educational status, in different age groups and different regions, and who received advice from doctors or not (*p*<0.05) (Tables 2, 3, 4 and 5).

**Table 2:** Differences in knowledge scores and rate among women with different educational levels (n = 308) (From ANOVA and Chi-square test).

Educational level	Total Knowledge		Risk Factors of CC		CC prevention		Clinical symptoms of CC		Benefits of screening		Understanding of positive results	
	Score	KR	Score	KR	Score	KR	Score	KR	Score	KR	Score	KR
	X±SD	n(%)	X±SD	n(%)	X±SD	n(%)	X±SD	n(%)	X±SD	n(%)	X±SD	n(%)
Junior college or higher	19.11±6.85 ^b^ ^c^	10(55.6) ^a^ ^b^ ^c^	3.56±1.54 ^a^ ^b^ ^c^	6(33.3) ^c^	4.00±1.82 ^a^ ^b^ ^c^	8(44.4) ^c^	4.44±2.62 ^a^	8(44.4) ^c^	2.00±1.08 ^a^	12(66.7)	5.11±1.84 ^a^	6(33.3)
Senior/Technical school	15.06±7.77^d^	6(18.8) ^a^	1.94±1.70 ^a^ ^d^	4(12.5)	2.06±2.29 ^a^ ^d^	6(18.8) ^d^	3.50±2.59 ^d^	8(25.0) ^d^	1.44±1.34	18(56.3)	6.13±1.86 ^d^	16(50.0) ^d^
Junior high school	14.16±7.93^b^	40(22.7) ^b^ ^e^	1.93±1.97 ^b^ ^e^	28(15.9) ^e^	2.01±2.11 ^b^ ^e^	34(19.3)^e^	3.02±2.42 ^e^	38(21.6) ^e^	1.74±1.22 ^e^	104(59.1)	5.45±2.47 ^e^	102(58.0) ^e^
Primary school or illiteracy	7.61±5.67^c^ ^d^	4(4.9) ^c^ ^e^	0.71±1.28^a^ ^d^ ^e^	4(4.9) ^c^ ^e^	0.63±1.17 ^a^ ^d^ ^e^	2(2.4) ^c^ ^d^ ^e^	1.76±1.19 ^a^ ^d^ ^e^	2(2.4) ^c^ ^d^ ^e^	1.05±1.20 ^a^ ^e^	36(43.9)	3.46±2.30 ^a^ ^d^ ^e^	14(17.1) ^d^ ^e^
*p*	<0.001	<0.001*	<0.001	0.005*	<0.001	<0.001*	<0.001	<0.001*	<0.001	0.099	<0.001	-

KR: Knowledgeable Rate;

^**a**^, indicates a statistically significance significant difference between junior college or higher and senior/technical school;

^**b**^ indicates a statistically significance significant difference between junior college or higher and junior high school;

^**c**^ indicates a statistically significance significant difference between junior college or higher and primary school or illiteracy;

^**d**^ indicates a statistically significance significant difference between senior/technical school and primary school or illiteracy;

^**e**^ represent there is a statistical significance between junior high school and primary school or illiteracy.

For ANOVA test, *p* <0.05 indicates a statistically significant difference between subgroups; for Chi-square test, *p* <0.0125 indicates a statistically significant difference between subgroups.

* Fisher’s Exact Test

**Table 3:** Differences in knowledge scores across disparate regions (n = 308) (From ANOVA and Chi-square test).

Regions	Total Knowledge		Risk Factors of CC		CC prevention		Clinical symptoms of CC		Benefits of screening		Understanding of positive results	
	Score	KR	Score	KR	Score	KR	Score	KR	Score	KR	Score	KR
	X±SD	n(%)	X±SD	n(%)	X±SD	n(%)	X±SD	n(%)	X±SD	n(%)	X±SD	n(%)
Most developed	19.38±8.19^a^ ^b^	48(50.0)^a^ ^b^	3.00±2.13 ^a^ ^b^	34(35.4) ^a^ ^b^	3.56±2.07 ^a^ ^b^	40(417) ^a^ ^b^	4.771±2.67 ^a^ ^b^	46(47.9) ^a^ ^b^	2.15±1.20 ^a^ ^b^	70(72.9) ^a^ ^b^	5.90±2.31 ^a^ ^b^	58(60.4) ^b^
Developed	11.87±6.34^a^ ^c^	12(11.35)^a^ ^c^	1.40±1.66^a^ ^c^	8(7.5) ^a^ ^c^	1.49±1.83 ^a^ ^c^	10(9.4) ^a^ ^c^	2.26±1.73 ^a^ ^c^	10(9.4) ^a^ ^c^	1.57±1.18 ^a^ ^c^	60(56.6) ^a^ ^c^	5.15±2.45 ^a^ ^c^	50(47.2) ^c^
Less developed	7.77±4.55^b^ ^c^	0(0.0) ^b^ ^c^	0.83±1.08 ^b^ ^c^	0(0.0) ^b^ ^c^	0.42±0.69 ^b^ ^c^	0(0.0) ^b^ ^c^	1.60±0.90 ^b^ ^c^	0(0.0) ^b^ ^c^	0.96±1.12 ^b^ ^c^	40(37.7) ^b^ ^c^	3.960±2.40 ^b^ ^c^	30(28.3) ^b^ ^c^
*p*	<0.001	<0.001	<0.001	<0.001	<0.001	<0.001	<0.001	<0.001	<0.001	<0.001	<0.001	<0.001

KR: Knowledgeable Rate;

^**a**^, indicates a statistically significance significant difference between most developed and developed regions;

^**b**^ indicates a statistically significance significant difference between most developed and less developed regions;

^**c**^ indicates a statistically significance significant difference between developed and less developed regions.

For ANOVA test, *p*<0.05 indicates a statistically significant difference between subgroups; for Chi-square test, *p*<0.0167 indicates a statistically significant difference between subgroups.

**Table 4:** Differences in knowledge scores among women who received advice from doctors or not (n = 308) (From *t*-test).

Received advice from doctors	Total Knowledge		Risk Factors of CC		CC prevention		Clinical symptoms of CC		Benefits of screening		Understanding of positive results	
	Score	KR	Score	KR	Score	KR	Score	KR	Score	KR	Score	KR
	X±SD	n(%)	X±SD	n(%)	X±SD	n(%)	X±SD	n(%)	X±SD	n(%)	X±SD	n(%)
Yes	15.66±8.09	38(30.6)	2.27±2.10	28(22.6)	2.45±2.23	32(25.8)	3.58±2.44	34(27.4)	1.94±1.17	84(67.7)	5.42±2.33	66(53.2)
No	10.87±7.36	22(12.0)	1.32±1.63	14(7.6)	1.30±1.78	18(9.8)	2.30±2.06	22(12.0)	1.27±1.25	86(46.7)	4.67±2.58	72(39.1)
***p***	<0.001	<0.001	<0.001	<0.001	<0.001	<0.001	<0.001	0.001	0.006	<0.001	0.010	0.015

KR: Knowledgeable Rate; P<0.05 indicates a statistically significant difference between subgroups

**Table 5:** Differences in Knowledge Scores and Rate among women of different age groups (n = 308) (From ANOVA and Chi-square test).

Age group	Total Knowledge		Risk Factors of CC		CC prevention		Clinical symptoms of CC		Benefits of screening		Understanding of positive results	
	Score	KR	Score	KR	Score	KR	Score	KR	Score	KR	Score	KR
	X±SD	n(%)	X±SD	n(%)	X±SD	n(%)	X±SD	n(%)	X±SD	n(%)	X±SD	n(%)
<34	15.37±5.15 ^c^	6(18.8)	2.06±1.81	4(12.5)	2.13±1.93	6(18.8)	2.69±1.79	4(12.5)	2.38±0.94^a^ ^b^ ^c^	26(81.3) ^b^	6.13±1.93 ^a^ ^b^ ^c^	24(75.0) ^a^ ^b^ ^c^
35–44	13.23±8.17	24(22.6)	1.81±2.01	16(15.1)	1.85±2.13	20(18.9)	2.98±2.46	24(22.6)^e^	1.70±1.23 ^a^	66(62.3) ^c^ ^d^	4.89±2.46 ^a^	42(39.6) ^a^
45–54	12.79±8.56	26(20.6)	1.71±1.91	20(15.9)	1.84±2.21	22(17.5)	3.00±2.46	26(20.6)	1.32±1.28^b^	56(44.4) ^b^ ^d^	4.92±2.46 ^b^	56(44.4) ^b^
55–64	9.91±6.98 ^c^	4(9.1)	1.14±1.47	2(4.5)	1.09±1.46	2(4.5)	2.00±1.56	2(4.5) ^e^	1.18±1.17^c^	22(50.0) ^c^	4.50±2.95 ^c^	16(36.4) ^c^
*p*	0.024	0.289	0.140	0.253*	0.113	0.154	0.072	0.044	<0.001	0.001	0.036	0.003*

**KR**: Knowledgeable Rate.

^**a**^ indicates a statistically significant difference between age groups <34 and 35–44;

^**b**^ indicates a statistically significant difference between age groups <34 and 45–54;

^**c**^ indicates a statistically significant difference between age groups <34 and 55–64;

^**d**^ indicates a statistically significant difference between age groups 35–44 and 45–54;

^**e**^ indicates a statistically significant difference between age groups 35–44 and 55–64.

For ANOVA test, *p* <0.05 indicates a statistically significant difference between subgroups; for Chi-square test, *p* <0.0125 indicates a statistically significant difference between subgroups.

* Fisher’s Exact Test

### Risk Factors of CC

The mean knowledge score for the risk factors of CC among all respondents was 1.70±1.89, out of a possible range of 0 to 7. The knowledge rate of risk factors of CC was only 13.6%. More than one third of the respondents (37.0%) didn’t know the risk factors of CC. Most of them only recognized one (21.4%) or two (14.3%) risk factors. The highest proportion of correct answers for risk factors was about “had sexual intercourse and children at a young age” (37.7%), followed by “having multiple partners or partners who have multiple partners” (30.5%), “history of sexually transmitted diseases” (25.3%), “aged 30–65 years” (24.7%) and “hormonal contraceptive use” (21.5%). Only 54(17.5%) and 40(13.0%) of the respondents regarded “HPV infection” and “smoking” as risk factors of CC (Table 6).

**Table 6:** Frequency of correct answer for items about CC among women in different regions (n = 308) (From Chi-square test).

Items	Most developed regions (N = 96)	Developed regions (N = 106)	Less developed regions (N = 106)	Total frequency	*χ* ^2^	*p*
**Section 1 Risk factors of CC**						
Having multiple partners or partners who have multiple partners	56(58.3)	30(28.3)	8(7.5)	94(30.5)	61.649	<0.001
Had sexual intercourse and children at a young age	54(56.3)	24(22.6)	38(35.8)	116(37.7)	24.463	<0.001
History of Sexually transmitted diseases	54(56.3)	22(20.8)	2(1.2)	78(25.3)	80.510	<0.001
History of HPV infection	34(35.4)	6(5.7)	0(0.0)	40(13.0)	63.595	<0.001
Aged 30–65	40(41.7)	24(22.6)	12(11.3)	76(24.7)	25.319	<0.001
Hormonal contraceptive use	40(41.7)	18(17.0)	8(7.5)	66(21.5)	36.730	<0.001
Smoking	10(10.4)	24(22.6)	20(18.9)	54(17.5)	5.406	0.067
**Section 2 CC prevention**						
Reduce numbers of sexual partners	54(56.3)	20(18.9)	4(3.8)	78(25.3)	76.919	<0.001
Late marriage and late childbirth	48(50.0)	6(5.7)	2(1.9)	56(18.2)	95.426	<0.001
Using condom	66(68.8)	14(13.2)	0(0.0)	80(26.0)	137.536	<0.001
No smoking	12(12.5)	22(20.8)	4(3.8)	38(12.3)	14.134	0.001
Having HPV vaccine before sexual debut	26(27.1)	2(1.9)	0(0.0)	28(9.1)	54.861	<0.001
Prompt treatment of STIs	66(68.8)	36(34.0)	0(0.0)	102(33.1)	107.551	<0.001
CC screening	70(72.9)	58(54.7)	34(32.1)	162(52.6)	33.993	<0.001
**Section 3 Symptoms of CC**						
Post-menopausal bleeding	58(60.4)	16(15.1)	4(3.8)	78(25.3)	94.412	<0.001
Abnormal vaginal discharge	62(64.6)	32(30.2)	12(11.3)	106(34.4)	64.595	<0.001
Vulvar itching or burning sensation (Choose “No”)	48(50.0)	82(77.4)	106(100.0)	236(76.6)	70.360	<0.001
Inter-menstrual bleeding	62(64.6)	22(20.8)	16(15.1)	100(32.5)	66.383	<0.001
Vaginal discharge	32(33.3)	10(9.4)	0(0.0)	42(13.6)	49.952	<0.001
Pelvic pain	40(41.7)	24(22.6)	0(0.0)	64(20.8)	53.470	<0.001
Bleeding after sexual intercourse, douching, or a pelvic exam	70(72.9)	20(18.9)	12(11.3)	102(33.1)	101.106	<0.001
Urinary frequency, urgency	30(31.3)	8(7.5)	0(0.0)	38(12.3)	48.916	<0.001
Longer or heavier menstrual periods	56(58.3)	26(24.5)	20(18.9)	102(33.1)	40.806	<0.001
**Section 4 Benefits of screening**						
Early detection of cervical precancerous lesions or cancer	74(77.1)	68(64.2)	48(45.3)	190(61.7)	21.970	<0.001
Early diagnosis of cervical precancerous lesions or cancer	60(62.5)	30(28.3)	12(11.3)	102(33.1)	61.264	<0.001
Early treatment of cervical precancerous lesions or cancer	72(75.0)	68(64.2)	42(39.6)	182(59.1)	27.794	<0.001
**Section 6 Understanding of the positive results**						
Negative screening result means cervix without any lesion, needing no more screening (Choose “No”)	78(81.3)	82(77.4)	52(49.1)	212(68.8)	29.814	<0.001
Negative screening result means cervix without any lesion, but still needing screening every 2–3 years	62(64.6)	66(62.3)	44(41.5)	172(55.8)	13.578	0.001
Positive screening result means suffering from CC(Choose “No”)	50(52.1)	38(35.8)	12(11.3)	100(32.5)	39.019	<0.001
Positive screening result means suffering from early stage cervical carcinoma (Choose “No”)	48(50.0)	34(32.1)	10(9.4)	92(29.9)	39.949	<0.001
Positive screening result means suffering from cervical precancerous lesions (Choose “No”)	44(45.8)	18(17.0)	8(7.5)	70(22.7)	45.086	<0.001
Positive screening result means there is cervical lesion, it needs further diagnosis	76(79.2)	64(60.4)	40(37.7)	180(58.4)	35.853	<0.001
CC is an incurable disease (Choose “No”)	70(72.9)	90(84.9)	88(83.0)	248(80.5)	5.260	0.072
CC is a disease can be treated	82(85.4)	86(81.1)	84(79.2)	252(81.8)	1.341	0.511
CC is curable disease	56(58.3)	68(64.2)	82(77.4)	206(66.9)	8.777	0.012

Analyses by Chi-square, ANOVA test and *t*-test revealed statistically significant differences in knowledge rate and score of risk factors of CC among women with different educational levels, in different regions, and who received advice from doctors or not (***p***<0.001) (Tables 2, 3 and 4).

### CC prevention

The majority of the participants (280, 90.9%) considered that CC could be prevented. However, the average total knowledge score for prevention measures of CC among all participants was only 1.77±2.07, out of a possible range of 0 to 7. The knowledge rate for prevention measures of CC was only 16.2%. About 40% of participants were unaware of prevention measures of CC. Most of them (76, 27.4%) only knew one of the prevention measures.

Over half of the participants knew that “CC screening” (162, 52.6%) could prevent CC occurring. However, the proportions of correct answers about other prevention measures were low. Out of 308 respondents 102 (33.1%) believed “prompt treatment of STIs” was one of the CC prevention measures; 80 (26.0%), 78(25.3%) and 56(18.2%) believed “using condoms”, “reducing number of sexual partners” and “late marriage and late childbirth”, respectively, could prevent CC occurring. Only 38(12.3%) and 28(9.1) knew “no smoking” and “having HPV vaccine before sexual debut” were also CC prevention measures (Table 6).

The results of the Chi-square, ANOVA test and *t*-test revealed statistically significant differences in the knowledge rate and score for CC prevention measures among women with different educational levels, in different regions, and who received advice from doctors or not (***p***<0.001) (Tables 2, 3 and 4).

### Clinical symptoms of CC

The mean total knowledge score for symptoms of CC among all participants was 2.82±2.304, out of a possible range of 0 to 9. The knowledge rate for clinical symptoms of CC was only18.2%. The majority of the respondents (99.4%) knew one of the clinical symptoms of CC. However, 61.0% of them only knew two or more of the symptoms.

About 30% of participants correctly reported that “abnormal vaginal discharge” (106, 34.4%), “bleeding after sexual intercourse, douching, or a pelvic exam” (102, 33.1%), “longer or heavier menstrual periods” (102, 33.1%) and “inter-menstrual bleeding” (100, 32.5%) were symptoms of CC. About 20% could correctly report that “post-menopausal bleeding (78, 25.3%), “pelvic pain” (64, 20.8%) were symptoms of CC. However, few of the participants knew that CC patients could also experience the following symptoms “vaginal discharge” (42, 13.6%), “urinary frequency, urgency” (38, 12.3%). Indeed, almost a quarter (23.4%) of the respondents wrongly regarded “vulvar itching or burning sensation” as one of the symptoms of CC (Table 6).

The results of the Chi-square test showed that a statistically significant difference was identified in the knowledge rate for CC clinical symptoms among women in different age groups (***p***<0.05) (Table 2). The results of the Chi-square, ANOVA test and *t*-test also revealed statistically significant differences in the knowledge rate and score for CC clinical symptoms among women with different educational levels, in different regions, and who received advice from doctors or not (***p***<0.001) (Tables 2, 3 and 4).

### Benefits of screening

The mean total knowledge score for benefits of screening among all participants was 1.54±1.26, out of a possible range of 0 to 3. The knowledge rate for benefits of screening was 55.2%. Most participants believed that CC screening could enable “early detection of cervical precancerous lesions or cancer” (190, 61.7%) and “early treatment of cervical precancerous lesions or cancer” (182, 59.1%). However, only 102(33.1%) participants believed that CC screening could enable “early diagnosis of cervical precancerous lesions or cancer” (Table 6).

The results of the Chi-square, ANOVA test and *t*-test showed statistically significant differences in the knowledge rate and scores for benefits of screening among women with different educational levels, in different age groups and regions, and who received advice from doctors or not (***p***<0.001) (Tables 2, 3, 4 and 5).

### Understanding of the positive results

The average total knowledge score for understanding about the positive results of CC screening among all participants was 4.97±2.51, out of a possible range of 0 to 9. The knowledge rate for understanding of the positive results was 44.8%. The majority of participants correctly answered that “CC is a curable disease” (252, 81.8%), “early CC can be cured” (206, 66.9%), “a positive screening result means there is cervical lesion, it needs further diagnosis” (180, 58.4%), and “negative screening result means cervix without any lesion, but still needing screening every 2–3 years” (172, 55.8%). However, many women mistakenly believed or were not sure that “negative screening result means cervix without any lesion, needing no more screening” (96, 31.2%), “positive screening result means suffering from CC” (208, 67.5%), “positive screening result means suffering from cervical precancerous lesions” (238, 77.3%), “positive screening result means suffering from early stage cervical carcinoma” (216, 70.1%) and “CC is an incurable disease” (60, 19.5%) (Table 6).

The results of Chi-square, ANOVA test and *t*-test showed statistically significant differences in the knowledge rate and scores for understanding of the positive results among women with different educational levels, in different age groups and regions, and who received advice from doctors or not (***p***<0.05) (Tables 2, 3, 4 and 5).

A logistic regression analysis was used to further analyze the statistical difference factors associated with total and sectional knowledge rates, with “unknowledgeable” as the reference group. The results showed that there was a positive association between “knowledgeable” (>60% correct answers regarding CC and clinical symptoms) and living in most developed or developed regions, receiving advice from doctors, higher levels of education and young women. There was also a positive relation between “knowledgeable” regarding risk factors, prevention and benefits of screening and living in most developed or developed regions, receiving advice from doctors, and young women. Living in most developed or developed regions was also a protective factor for “knowledgeable” of understanding of the positive results of CC screening. (Table 7)

**Table 7:** Logistic Regression Analysis of Factors Associated with total and sectional knowledge rate (N = 308).

Influence factors	B	Wald	*P*	OR	95%CI
***Total knowledge***					
Regions ^a^	3.269	38.850	0.000	26.279	9.402~73.452
Receive advice from doctors ^b^	1.547	14.323	0.000	4.696	2.108~10.462
Education ^c^	0.490	3.550	0.060	1.632	0.980~2.718
Age	-0.061	5.055	0.025	0.941	0.892~0.992
***Risk Factors of CC***					
Regions ^a^	3.061	28.760	0.000	21.342	6.973~65.318
Receive advice from doctors ^b^	1.424	11.349	0.001	4.154	1.814~9.513
Education ^c^	.090	0.110	0.740	1.095	0.641~1.868
Age	-.071	5.672	0.017	0.931	0.878~0.987
***CC prevention***					
Regions ^a^	3.217	32.297	0.000	24.949	8.227~75.659
Receive advice from doctors ^b^	1.321	10.608	0.001	3.747	1.692~8.296
Education ^c^	0.480	3.452	0.063	1.616	0.974~2.683
Age	-0.079	7.171	0.007	0.924	0.873~0.979
***Clinical symptoms of CC***					
Regions ^a^	3.475	37.926	0.000	32.311	10.690~97.661
Receive advice from doctors ^b^	1.289	10.169	0.001	3.628	1.643~8.011
Education ^c^	0.576	4.959	0.026	1.778	1.071~2.951
Age	-0.824	36.816	0.006	0.439	0.243~0.790
***Benefits of screening***					
Regions ^a^	0.754	20.977	0.000	2.125	1.539
Receive advice from doctors ^b^	0.748	8.610	0.003	2.113	1.282
Education ^c^	-0.056	0.106	0.745	0.945	0.675~1.325
Age	-0.033	5.104	0.024	0.967	0.939~0.996
***Understanding of the positive results***					
Regions ^a^	0.622	14.823	0.000	1.862	1.357~2.555
Receive advice from doctors ^b^	0.430	3.032	0.082	1.538	0.947~2.496
Education ^c^	0.184	1.166	0.280	1.202	0.861~1.678
Age	-0.024	2.687	0.101	0.977	0.949~1.005

^a^:0 = less developed, 1 = developed, 2 = most developed;

^b^:0 = no,1 = yes;

^c^:0 = primary school or illiteracy, 1 = junior high school, 2 = senior/Technical school, 3 = junior college or higher

## Discussion

This study is the first to explore the knowledge that women have about CC screening in project counties of NCCPRA across different socio-economic regions of China. The most important finding from this study data indicates that women’s knowledge about CC in project counties was inadequate. Although all of the respondents in this study had participated in the NCCPRA and received the free CC screening service, still 6.5% of the women had never heard about CC. The total knowledge rate of CC was only 19.5%, which is consistent with the study conducted in Nigeria, where only 11.8% of the rural women and 17.6% of urban women had knowledge of CC [18].

The majority of the respondents were able to identify at least one risk factor for CC, which is consistent with the study conducted in rural areas of South Africa (64%) [23], but higher than the findings from Ethiopia (31.0%) [25] and Uganda (29%) [24]. The difference might be attributed to the fact that South Africa also has a national CC screening program, but in the other countries, there was no such program. In this study, women were more aware of the risks associated with having sexual intercourse and children at a young age, having multiple partners or partners who have multiple partners, having history of STIs, aged 30–65 years and hormonal contraceptive use. However, a worrisome finding in the study was that HPV infection and smoking were not recognized as risk factors by the majority of participants. It was much lower than the results from Malaysia [28], Turkey [17], and Singapore [27], which showed that more than 50% of the participants were aware of that smoking and HPV infection were related with CC. This may be explained by the fact that smoking is not a common practice among Chinese women (the prevalence of smoking is only 2.4% among women) [29], smoking is most commonly associated with lung cancer, and HPV vaccine is not available in China. It was troubling that many studies had identified that women were unaware of the link between infection with HPV and CC, which could place them at greater risk of contracting CC [30–32]. Thus, it is very important to emphasis the high HPV prevalence and the link between smoking and CC when providing health education to women.

A worrisome finding in this study is that about 40% of the respondents did not know that CC can be prevented. Over half of the participants recognized CC screening as a prevention measure for CC, which is lower than the study done in Ethiopia (63.9%) [25] and South Africa (57.0%) [23]. Although prompt treatment of STIs, using condoms, reducing the number of sexual partners, late marriage and childbirth, no smoking and HPV vaccine were recognized as prevention measures by only 10.0%-30.0% of participants, the correct proportions were still higher than the study conducted among women in Ethiopia (less than 6.0%) [25]. The lower correct proportions of recognizing no smoking (12.3%) and HPV vaccine (9.1%) as prevention measures may be explained by the fact that there were lower correct proportions of recognizing smoking (13.0%) and HPV infection (17.5%) as risk factors for CC.

Although almost 100.0% of the respondents knew the clinical symptoms of CC, the correctly reporting proportions of different symptoms were less than 30.0%. This result contrasts with a previous study in Turkey [17] where the correct proportions were about 50.0%. A possible explanation for this difference may be due to the fact that the recruited women in Turkey were gynecology clinic patients. Most of them likely visited doctors for clinical symptoms, whereas in this study, the women represented a healthy population without any symptoms.

A positive finding in this study was that 81.8% of the respondents believed that CC was a curable disease, which is consistent with the previous study conducted in rural areas of China (80.8%) [15] and higher than the study done in Turkey [17]. It was troubling that about 70.0% of the respondents incorrectly believed that positive screening result meant suffering from CC (67.5%), cervical precancerous lesions (77.3%), or early stage cervical carcinoma (70.1%). This pessimistic attitude towards positive screening results and CC can make women more frightened of CC screening, and exhibit a characteristic of fatalism [17]. This may be a barrier to subsequent participation in cancer screening [15, 33].

Our study also found there was a significant association between the age, educational status, regional socio-economic, and doctor’s advice and the knowledge about CC. The women who were younger (<34 years old), with higher education (junior college or higher degree), in most developed regions and who had received advice from doctors gave more correct answers to the questions in this study. Significantly lower knowledge scores were seen in women who were older (55–64 years old), had lower education (with primary school degree or illiteracy), lived in less developed regions women and women who did not receive any advice for her screening results from doctors.

In this study, it is important to note that the regional socio-economic level was the key factor that influenced the knowledge level of CC among women. The results of logistic regression analysis showed that women living in most developed and developed regions were more likely to have a greater overall knowledge and a higher percentage of women had adequate knowledge about risk factors, prevention, clinical symptoms, benefits of screening, and understanding of the positive results than women living in less developed regions. This result also has been supported by another study on knowledge of breast cancer conducted in China, which showed that the total and sectional knowledge scores about breast cancer in the under-developed areas were also significantly lower than in developed areas [21].

Higher education level, receiving advice from doctors, and young age were also significant predictors of higher knowledge about CC screening. Previous studies showed similar results in which higher educational status [17, 18, 25, 34, 35], younger age group [17] and visiting a health institution [25] were statistically significantly associated with adequate knowledge.

The significant differences in different subgroups of this study could be explained by the fact that women who were in less developed regions had disadvantage compared with the other regions in terms of poorer education (56.6% with primary school or illiterate) and poorer access to health services (71.7% had never received the health advice from doctors). Younger women who were recruited in this study had higher educational level, and they were of reproductive age and participated in the routine procedures for prenatal care or family planning. Moreover, a previous study has shown that women who have visited a health institution have a higher chance of getting more comprehensive information [25].

The results of this research suggest that there are two key areas for improvement to enhance the CC knowledge of women eligible for screening. Firstly, a health promotion intervention should be developed to target older women, women with less education and women in less developed regions and focus on improving CC knowledge. This should also encourage women to talk to their doctor if they wish to find out more as this was found to be a positive predictor of CC knowledge. The second intervention should target health care providers as this communication appears to be very important and so their knowledge of CC needs to be accurate and up to date so that the best possible advice can be provided.

## Limitations

This study has several limitations. It only focused on the knowledge of the women in some project counties of NCCSPRA. It may not be generalized to all target populations of this program, especially the target women who didn’t attend screening, as all of the recruited participants had received the free CC screening services. Therefore, the results of this study should be interpreted with caution. This study used a cross-sectional design; thus, it only speculated on the causal relationship between the variables. It used convenience sampling, so the results might be unrepresentative of the population being studied. However, despite these limitations, the results of this study provide a basis for further planning future in-depth research prior to developing educational materials and planning training-based interventions for the implementation of the NCCSPRA.

## Conclusion

This is the first study to provide insight into the level of knowledge about CC among women in project counties of NCCSPRA in different socio-economic regions of China. The results of this study revealed that though all of the participants had received free CC screening services in this program, knowledge about CC was very poor among the participants especially among the older women, and women who had lower educational status and were in less developed regions of China. Thus, we can hypothesize that the knowledge of women who didn’t attend CC screening in the project counties or those living in non-project counties would be very low.

Findings from this study underscore the need to redesign the strategies of the national CC screening program to strengthen the health education in this program, not just provide screening services. It is urgent to create a targeted educational intervention to improve the women’s knowledge about CC in project counties. Such intervention would need to pay more attention to older women and those with low education level, especially in the less developed regions. In addition, educating, training and motivating healthcare providers to play a key role in providing knowledge to women is also a significant element of improving women’s knowledge level. The data from this study makes an important contribution to improving the understanding of links among the variables and offer useful elements for planning future in-depth research prior to developing educational materials and planning relative interventions for the implementation of the NCCSPRA.
